# Investigating the demographic history of Japan using ancient oral microbiota

**DOI:** 10.1098/rstb.2019.0578

**Published:** 2020-10-05

**Authors:** Raphael Eisenhofer, Hideaki Kanzawa-Kiriyama, Ken-ichi Shinoda, Laura S. Weyrich

**Affiliations:** 1 Australian Centre for Ancient DNA, University of Adelaide, Adelaide, Australia; 2 Department of Anthropology, National Museum of Nature and Science, Tsukuba, Japan; 3 Department of Anthropology and the Huck Institutes of Life Sciences, The Pennsylvania State University, University Park, PA, USA

**Keywords:** Japan, microbiome, ancient DNA, phylogenomics, palaeomicrobiology

## Abstract

While microbial communities in the human body (microbiota) are now commonly associated with health and disease in industrialised populations, we know very little about how these communities co-evolved and changed with humans throughout history and deep prehistory. We can now examine these communities by sequencing ancient DNA preserved within calcified dental plaque (calculus), providing insights into the origins of disease and their links to human history. Here, we examine ancient DNA preserved within dental calculus samples and their associations with two major cultural periods in Japan: the Jomon period hunter–gatherers approximately 3000 years before present (BP) and the Edo period agriculturalists 400–150 BP. We investigate how human oral microbiomes have changed in Japan through time and explore the presence of microorganisms associated with oral diseases (e.g. periodontal disease, dental caries) in ancient Japanese populations. Finally, we explore oral microbial strain diversity and its potential links to ancient demography in ancient Japan by performing phylogenomic analysis of a widely conserved oral species—*Anaerolineaceae* oral taxon 439. This research represents, to our knowledge, the first study of ancient oral microbiomes from Japan and demonstrates that the analysis of ancient dental calculus can provide key information about the origin of non-infectious disease and its deep roots with human demography.

This article is part of the theme issue ‘Insights into health and disease from ancient biomolecules’.

## 1. Introduction

Microbiota within the human body possess functions that can influence the development, physiology, behaviour and the health of their hosts [1–8]. Therefore, altering these functions can lead to disease, compromising the health of the host [9,10]. Microbiota alterations result from a range of factors, including the use of antibiotics [11,12], changes in diet [13], infection by pathogens [14,15] and the adoption of lifestyles associated with industrialization [16]. Evidence suggests that specific microbes within the microbiota can be vertically inherited [17–20] and have been co-speciating with humans throughout hominid evolution [21,22]. Consequently, different human populations can have distinct microbiota as a result of their unique evolutionary and demographic histories [16,23–25].

Understanding the factors that drive microbial variation and examining how these microbial communities have changed and adapted over time with humans can provide key insights into human health. However, little is known about how the human microbiome has adapted and evolved in human history, especially during cultural admixture (e.g. when Europeans first met the peoples of the Americas). Such cultural admixtures could disrupt long-term relationships between microbiomes and host, and potentially contribute to microbiome disturbances that could influence host health [26]. Additionally, microbial lineage replacement owing to cultural/population admixture could also shape the microbiome in distinct ways; for example, ‘signatures’ of past human interaction and population replacement (e.g. loss of particular species or strains) may still be present in living populations today [27].

Recently, ancient human calcified dental plaque (calculus) was identified as a robust source of ancient human-associated microbial DNA [28–31] and now allows researchers to examine human-associated microbial communities from the past. Dental calculus is the result of a microbial biofilm that grows on teeth and undergoes periodic mineralization events that lock oral microorganisms in place within a robust calcium phosphate matrix [32]. The direct association of dental calculus on human teeth, coupled with its robust nature, provides an unprecedented opportunity to examine the bioarchaeological record of past human oral microbiomes, allowing researchers to identify factors that have altered the oral microbiome through time [28–30]. For example, dental calculus research has correlated shifts in the European microbiota community composition to large-scale dietary and lifestyle changes (e.g. from hunting–gathering to an agricultural lifestyle in Europe) [28]. Dental calculus is, therefore, a tool that can be used to sample the oral microbiome of past human populations and explore how the microbiome adapts and evolves following major cultural and demographic shifts.

Japan is an ideal location to examine human-associated microbiota change and evolution, as Japan has experienced large shifts in diet, culture, and demography over time and is geographically isolated from mainland Asia. The Japanese Archipelago was largely inhabited by the Jomon culture from approximately 16 000 to 3000 years before present (BP) [33,34]. Agriculture-bearing migrants from continental Asia came to the Japanese Archipelago and admixed with the Jomon during the early Yayoi period around 3000 BP [35–37]. Both modern and ancient DNA studies suggest that the admixture was weighted towards migrants, with modern estimates of Jomon contribution to mainland Japanese populations being less than 20% [37,38]. Before this admixture, a mitochondrial divergence estimate suggests that over 22 000 years of separation existed between the Jomon and continental Asian populations [39], which, coupled with their putatively disparate lifestyles (e.g. hunter–gatherer versus agriculturalist), may have resulted in divergent coevolution of their microbiomes. Here, we examine bacterial DNA preserved within ancient dental calculus from the Jomon (approx. 3000 BP) and Edo periods (400–150 BP) in Japan to investigate how and why microbial communities changed in the past.

## 2. Methods

### (a) Ancient dental calculus samples

**Figure 1.**
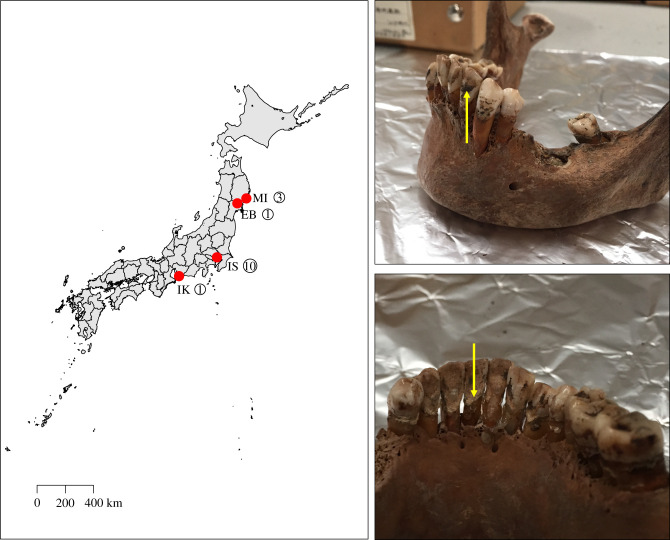
Map of Japan illustrating sites where ancient dental calculus samples were collected. EB, Ebishima; IK, Ikawazu; IS, Ikenohata Shichikencho, MI , Miyano. Yellow arrow in top right box denotes black teeth painting (Ohaguro). Yellow arrow in bottom right box denotes dental calculus.

Ancient dental calculus samples (5 = Jomon, approx. 2400–3000 BP; 10 = Edo, 400–150 BP) (figure 1) were collected from the Natural Museum of Nature and Science in Tsukuba, Ibaraki, Japan. Of the Jomon samples, one was from the Ebishima shell mound in Iwate prefecture [40]. One was from the Ikenohata Shichikencho site in Ikawazu, Aichi prefecture, with radiocarbon dates of associated skeletal remains being 2440–3070 cal BP. [41]. Three were from a site in Miyano, Iwate prefecture [42]. The Edo period samples originated from the Ikenohata-Shichikencho site [43], which is located in Taito-ku, Tokyo. The excavation of this site was undertaken between 1993 and 1995 and yielded about six hundred graves which belong to the period from the late seventeenth to the nineteenth centuries [43]. The graves represented samurai and townsmen, known from the fact that the burials contained ceramic coffins (kamekan) and wooden coffins (mokkan) that were used for samurai and commoners, respectively.

Dental calculus was removed from specimens as previously described [44]. Briefly, a sterile dental pick was used to carefully remove dental calculus from one side of one tooth, and the specimen was placed in a sterile plastic bag for transport at room temperature to the Australian Centre for Ancient DNA at the University of Adelaide. Accompanying metadata was also collected at this time (electronic supplementary material, table S1).

### (b) DNA extraction and library preparation

As authentic ancient DNA can be contaminated by modern DNA, steps to minimize and monitor the introduction of contaminant DNA were used [45]. All sample processing and molecular biology procedures prior to polymerase chain reaction (PCR) amplification were carried out at the Australian Centre for Ancient DNA facility at the University of Adelaide. These experiments were performed within a specialized ancient DNA laboratory, which maintains positive air pressure, HEPA filtered air, daily ultraviolet (UV)-treatment, regular 3% bleach cleanings, and work in isolated still-air hoods located in isolated rooms to limit the introduction of modern contaminant DNA. All technicians entered the facility using a dedicated entry room and wore full-body clean suits, gloves, and facemasks. Dental calculus samples were decontaminated to minimize environmental contamination by UV-irradiation for 15 min on each side, following by soaking in 2 ml of 5% sodium hypochlorite for 3 min, rinsing in 80% ethanol for 1 min, and drying at room temperature for 2 min. Immediately post-decontamination, dental calculus samples were crushed on the side of plastic tubes with sterile tweezers, and DNA was extracted using an in-house silica-based method described previously [46]. Extraction blank controls were included to monitor microbial DNA background signals throughout this process; one extraction blank control was analysed for each DNA extraction batch (1 control: 10 samples).

Shotgun metagenomic libraries were constructed as previously described [47], using unique combinations of 7 bp forward and reverse barcodes. Thirteen cycles of PCR were used for the first amplification with P5/P7 barcoded adapters (Platinum™ *Taq* HiFi Polymerase), followed by an additional 13 cycles for the addition of GAII-index and sequencing primers. Metagenomic shotgun libraries were cleaned using Ampure XP, quantified using an Agilent TapeStation, and pooled at equimolar concentrations prior to initial sequencing on the Illumina NextSeq (2 × 150 bp), and further sequencing on an Illumina HiSeq (2 × 150 bp). All samples were sequenced together in the same sequencing pool.

### (c) Data used from other previously published studied

Eighteen modern dental plaque samples from the Human Microbiome Project (HMP) [48] were downloaded (SRS011098, SRS011126, SRS011152, SRS011255, SRS011343, SRS012285, SRS013170, SRS013252, SRS013533, SRS013723, SRS013836, SRS013949, SRS014476, SRS014578, SRS014690, SRS014894, SRS015044, SRS015063). Because MALT does not have a paired-end alignment mode, only the R1 files were used. The R1 files were randomly subsampled to a depth of 1 500 000 sequences using Seqtk with a seed of 666 https://github.com/lh3/seqtk. Modern and ancient dental calculus DNA sequences were obtained from a previous study [30] (https://www.oagr.org.au/experiment/view/65/).

### (d) Data processing and taxonomic composition analyses

The resulting data converted into FASTQ format using Illumina's bcl2fastq software, before being demultiplexed, trimmed and merged using AdapterRemoval 2 based on unique P5/P7 barcodes [49]. Only merged reads were used for subsequent analyses. Merged reads from separate sequencing runs were concatenated per sample. Taxonomic composition was determined using MEGAN Alignment Tool (MALTn) v. 0.3.8 [50], whereby DNA reads from samples were aligned (default settings) against the RefSeqGCS database (2017) [51] containing 47 713 archaeal and bacterial genome assemblies from the NCBI Assembly database [52]. The resulting blast-text files were converted into RMA files via the blast2rma script included in the program MEGAN v.6.11.1 [53], with the following lowest common ancestor (LCA) parameters: weighted-LCA = 80%, minimum bitscore = 44, minimum E-value = 0.01, minimum support per cent = 0.1 [51].

Samples were first assessed for ancient DNA authenticity by comparison to extraction blank controls. Subtractive filtering was used to remove species found in the extraction blank controls from ancient dental calculus samples. For analysis in QIIME [54], the filtered, species-level taxonomic composition was exported from MEGAN into BIOM format, and imported into QIIME 1.9.1 and rarefied to 86 267 species-level reads per sample. SourceTracker (v.0.9.8) analysis [55] was also carried out on this rarefied BIOM table to help examine exogenous contamination using various, previously published well-characterized source sample types: soil [56], skin [57,58], gut [25,59,60], saliva [61,62], dental plaque [63] and ancient dental calculus [30,31] (see the electronic supplementary material, table S7 for more information and accession numbers). Raw sequences from these studies were downloaded and processed in the same manner as the samples in the present study. PERMANOVA was used to test for statistical significance in composition between groups using the compare_categories.py script with 999 permutations. Differential abundance of species between groups was tested using the Kruskal–Wallis test with Bonferroni-correction in the group_significance.py script. The rarefied table was imported into STAMP [64] to calculate and plot the Welch's *t*-test of *Methanobrevibacter oralis* relative abundance (figure 4).

### (e) Whole-genome phylogenetic analysis

Genomic sequences were assembled by mapping reads to the *Anaerolineaceae* sp*. oral taxon 439* genome (RefSeq accession: GCF_001717545.1) using BWA-aln [65] with the seed disabled, as recommended for ancient DNA [66]. The resulting BAM files were filtered to remove reads with mapping quality of less than 1 (keeping reads that only have 1 best hit) using SAMtools [67], and duplicates were removed using DeDup [68]. Estimation of cytosine deamination was performed using MapDamage2 [69] using the *Anaerolineaceae* sp*. oral taxon 439* reference genome (RefSeq accession: GCF_001717545.1). Edit distances were calculated using BAMstats (https://github.com/guigolab/bamstats). Coverage visualizations were created using Anvi'o [70]. Samples with fewer than 100 000 mapped reads (electronic supplementary material, table S6) were excluded from phylogenetic analyses (A18017_Japan_Jomon_3, A18019_Japan_Jomon_5, and A18022_Japan_Edo_3). Variant calling was performed using the SNIPPY pipeline (https://github.com/tseemann/snippy), which uses FreeBayes [71]. The pipeline was adjusted to use a FreeBayes –ploidy of ‘1’. Using a .bed file, we masked 16S rRNA and tRNA gene regions and putative phage regions identified using PHASTER [72] (electronic supplementary material, file 5). To account for cytosine deamination, a minimum depth of 3 sequences was required to call a variant. A minimum fraction of 90% (i.e. greater than 90% of nucleotides at a site had to be the same) was used to ensure the dominant variant was used. Missing data (depth less than 3) were labelled as N's. Phylogenetic reconstruction was performed on the masked whole genome single nucleotide polymorphism alignment (.full.aln SNIPPY suffix) using RAxML [73], with the GTR-GAMMA substitution model and autoMRE bootstrapping (raxmlHPC-PTHREADS-SSE3 -f a -x 12345 -p 12345 -# autoMRE -m GTRGAMMA -s ‘alignment’ -o Elsidron1). Trees were visualized and annotated using FigTree (https://github.com/rambaut/figtree).

## 3. Results

### (a) Authentic ancient microbial DNA was isolated from dental calculus

**Figure 2.**
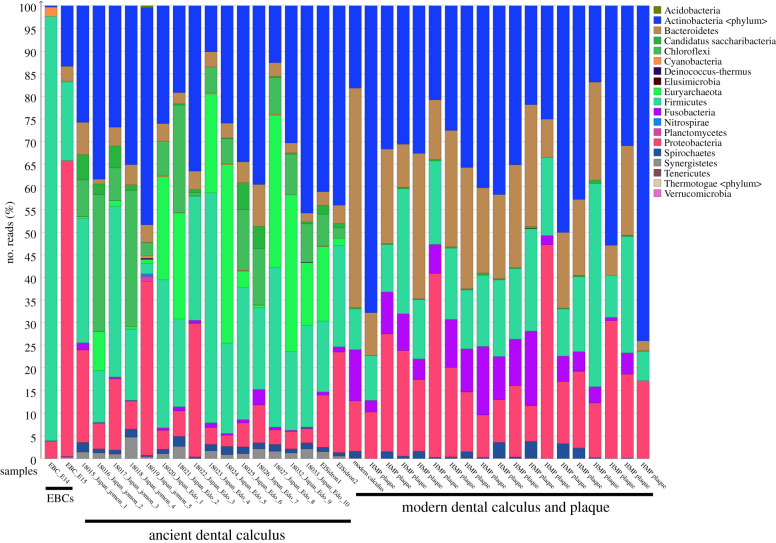
Phylum level taxonomic composition of samples and EBCs.

We applied metagenomic shotgun sequencing to 15 ancient Japanese dental calculus samples: five male Jomon period (approx. 3000 BP) and 10 (five male; five female) from the Early Edo period 400–300 BP) (figure 1). An average of 8 992 067 sequences per sample was obtained, with the fragment length distributions being as expected for ancient DNA (average size 78 bp; electronic supplementary material, table S2). We used MALTn (MEGAN Alignment Tool) to align DNA sequences to a reference database containing 47 713 archaeal and bacterial genome assemblies, and as expected for ancient dental calculus studies [51]; an average of 49.8% (±10.1%) of DNA sequences in each sample could be assigned taxonomy. The ancient Japanese calculus samples looked similar to previously published ancient calculus samples (figure 2) and were distinct from extraction blank controls (EBCs) (figure 2). Additionally, there were phyla present in the ancient calculus samples that were absent in modern plaque samples from the HMP and included Synergistetes, Chloroflexi, Candidatus Sacchararibacteria, and Euryarchaeota (figure 2). These phyla contain several species that can be associated with periodontal disease in modern populations, such as Synergistetes: *Fretibacterium fastidiosum* [74]; Chloroflexi: *Anaerolineaceae* sp*. oral taxon 439* [75]; Candidatus Sacchararibacteria: *TM7x* [76]; and Euryarchaeota: *Methanobrevibacter oralis* [77]. Therefore, the absence of these phyla from the modern plaque samples might be associated with disease-state, as all HMP samples were taken from healthy individuals [27], but a current lack of information regarding the microbiome in health status of ancient individuals makes this difficult to classify [78].

**Figure 3.**
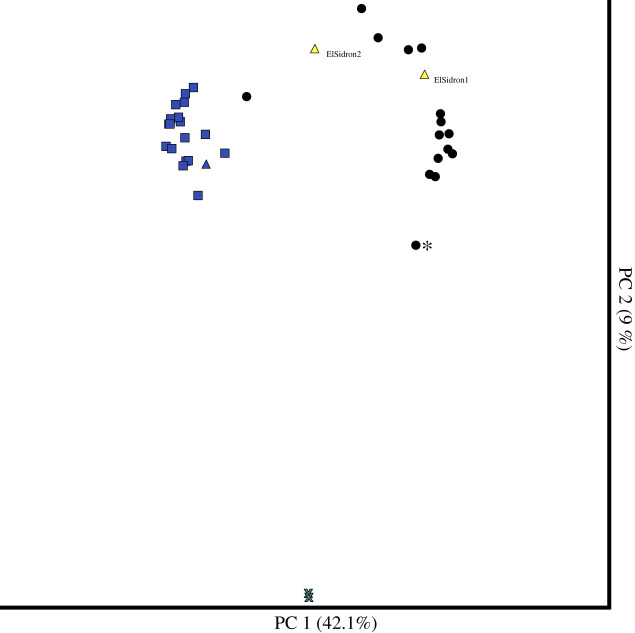
PCoA ordination of species-level Bray-Curtis distances. Extraction blank controls (EBCs, grey crosses), cluster separately from the rest of the ancient and modern oral samples. Ancient Japanese dental calculus samples (black circles) cluster with the El Sidron Neanderthal samples (yellow triangles). Modern dental plaque samples from the Human Microbiome Project (blue squares), and a modern dental calculus sample (blue triangle) cluster separately from ancient dental calculus samples, with the exception of one Edo period Japanese sample (18022_Japan_Edo_3). One Jomon sample (A18019_Japan_Jomon_5) is also pulled towards the EBCs (asterisk).

As background DNA contamination can influence ancient microbiome studies [27,79,80], we next assessed oral and contaminant DNA levels in the samples by ordinating Bray Curtis dissimilarity in a principal coordinates analysis (PCoA) (figure 3), which included EBCs, ancient Japanese samples, previously published ancient calculus specimens [30], and modern healthy plaque samples from the HMP [27]. Ancient Japanese calculus specimens clustered with published ancient calculus specimens and were dissimilar to EBCs, as expected (figure 3). Except for one Edo calculus specimen (A18022), ancient Japanese samples were distinct from modern plaque samples from the HMP (figure 3). We took a conservative approach and removed any species found in the EBCs (electronic supplementary material, table S3) from the Japanese calculus samples to help eliminate the contributions of contaminant DNA [81]; an average of 94.8% species-assigned sequences remained after filtering, highlighting the robust preservation of the specimens (electronic supplementary material, table S4). Species present in ancient Japanese samples after filtering by EBCs were largely previously identified in other oral microbiome studies [27,82] and have entries in the Human Oral Microbiome Database (HOMD) (electronic supplementary material, table S5), [83]. Lastly, we ran SourceTracker on the filtered dental calculus samples, which, of the source sample types used (skin, soil, gut, saliva, modern plaque and ancient dental calculus), predicted dental calculus was the most likely source, with the exception of sample A18022, which was predominantly modern plaque (electronic supplementary material, figure S8).

Upon closer taxonomic investigation of our samples, we noted that one sample (A18019_Japan_Jomon_5) was more contaminated and poorly preserved than the others. This sample was pulled towards the EBCs in the PCoA (figure 3) and had 8 out of 41 species classified that were of oral origin (HOMD) after filtering by EBCs (electronic supplementary material, figure S1). As samples with poorer preservation typically yield lower quantities of DNA, which can lead to higher percentages of duplicates, we assessed the percentage of duplicate sequences in this sample using BBMap's dedupe2 (sourceforge.net/projects/bbmap/) and found that 81.9% of sequences were duplicates. In the light of these findings, we removed this sample from subsequent compositional analyses.

### (b) Comparing the oral microbiota of Jomon and Edo periods Japan

As the Jomon and Edo cultures are associated with distinct diets and lifestyles, we wanted to explore the similarities and differences between the microbiomes found in both cultures. We found no significant differences in alpha diversity between Jomon and Edo period Japanese samples (Shannon and observed species non-parametric *t*-test *p*-values greater than 0.05), which probably reflects the difficulty in obtaining clear diversity signatures in ancient calculus specimens [84]. We also found no significant differences in composition (PERMANOVA of Bray-Curtis and Binary Jaccard distance *p*-values greater than 0.05) or differentially abundant species between the Edo period or Jomon samples (Kruskal–Wallis; Bonferroni-correction *p*-values greater than 0.05). However, one Edo period sample clustered closely with the modern dental plaque samples in figure 3 (A18022) and had 10 classified species that were not present in any other sample: *Rothia aeria, Corynebacterium durum, Actinomyces johnsonii, Actinomyces* sp*. HPA0247*, *Haemophilus parainfluenzae*, *Neisseria meningitidis*, *Neisseria sicca*, *Neisseria* sp*. HMSC072F04*, *Fusobacterium hwasookii* and *Porphyromonas* sp*. KLE 1280*.

### (c) Oral microbiota correlates with disease status and sex in the Edo period Japan

**Figure 4.**
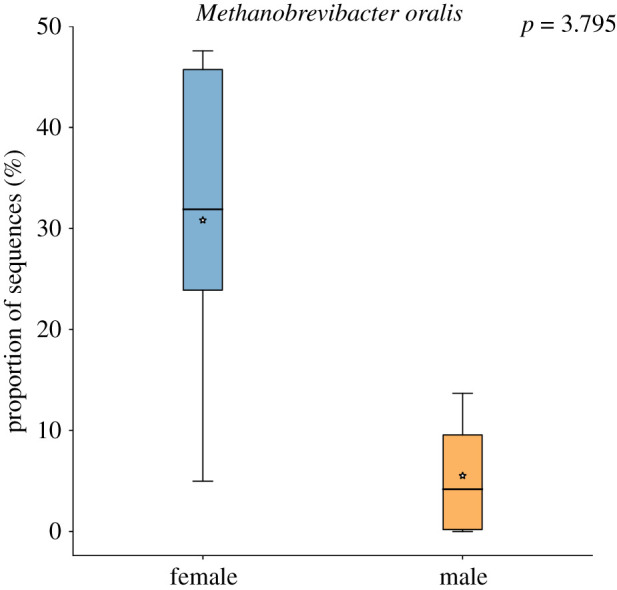
Box-plot of *Methanobrevibacter oralis* abundance in Edo period individuals. Females *n* = 5 (blue) versus males *n* = 5 (orange). Welch's *t*-test uncorrected *p*-value = 0.028, Benjamini–Hochberg false discovery rate corrected *p*-value 3.795.

All of the female specimens in this study had evidence of periodontal disease and had their teeth dyed black, which was a common cultural practice of females in the Edo period (called Ohaguro). Therefore, we wanted to test if the female samples had different microbiota to male samples in our study. Again, we found no significant differences in alpha diversity (Shannon and observed species *p*-values greater than 0.05), as expected. However, we did find a significant difference in microbiota composition (PERMANOVA of Bray–Curtis distances *p*-value 0.028, test statistic 2.35) in females versus males, although this was not observed in non-abundance weighted metric (PERMANOVA of Binary Jaccard *p*-value greater than 0.05). No species were significantly differentially abundant between males and females with periodontal disease (Kruskal–Wallis test with Bonferroni-correction *p*-values greater than 0.05). We also tested for signatures of periodontal disease [85–87] in female Edo individuals. No species were significantly associated with caries prevalence or periodontitis (Kruskal–Wallis test with Bonferroni-corrected *p*-values greater than 0.05), including members of the periodontitis-associated ‘red-complex’ (*Treponema denticola, Tannerella forsythia, Porphyromonas gingivalis*) [88]. However, the abundance of the periodontitis-associated archaeon, *Methanobrevibacter oralis* [77,89,90], was substantially higher in the females (mean abundance in females = 32%, mean abundance in males = 5%) (figure 4), although this difference was not statistically significant when controlling for multiple comparisons (Welch's t-test Benjamini-Hochberg false discovery rate corrected *p*-value 3.795). These results suggest that the oral microbiota composition in Japanese Edo women, who both practiced Ohaguro and suffered from periodontal disease, is distinct from Edo men.

### (d) Phylogenomic analysis

To further explore factors that drive microbial variation in ancient Japan, we performed phylogenomic analysis to explore strain diversity present in both periods. To find suitable candidates for phylogenomic analysis, we determined the core oral microbiome in ancient Japan (i.e. species present in every sample). We found *Actinomyces* sp*. oral taxon 414, Actinomyces dentalis, Anaerolineaceae* sp*. oral taxon 439,* and *Olsenella* sp*. oral taxon 807* to be present in all samples.

The oral bacterium *Anaerolineaceae* sp*. oral taxon 439* was chosen for phylogenetic analysis owing to its high mean relative abundance within calculus samples (16.5%), which yielded a greater depth of coverage and higher quality variant calls for our fairly low coverage sequencing data (electronic supplementary material, table S6). This bacterium is present at low abundance in healthy human plaque and higher abundance in individuals with periodontal disease [75]. This bacterium also has a high-quality, complete genome assembly needed for phylogenomic reconstructions, although it remains the only human-associated *Anaerolineaceae* genome currently publicly available. Sequences mapped against the *Anaerolineaceae* sp*. oral taxon 439* genome had terminal cytosine deamination typical of ancient DNA (electronic supplementary material, figures S2 and S3), with the sequences mapping from Jomon and El Sidron Neanderthal samples having higher levels of cytosine deamination at terminal ends (13.9%) compared to the more recent (400–150 year old) Edo samples (6%), as expected with the increasing age of the samples [91].

**Figure 5.**
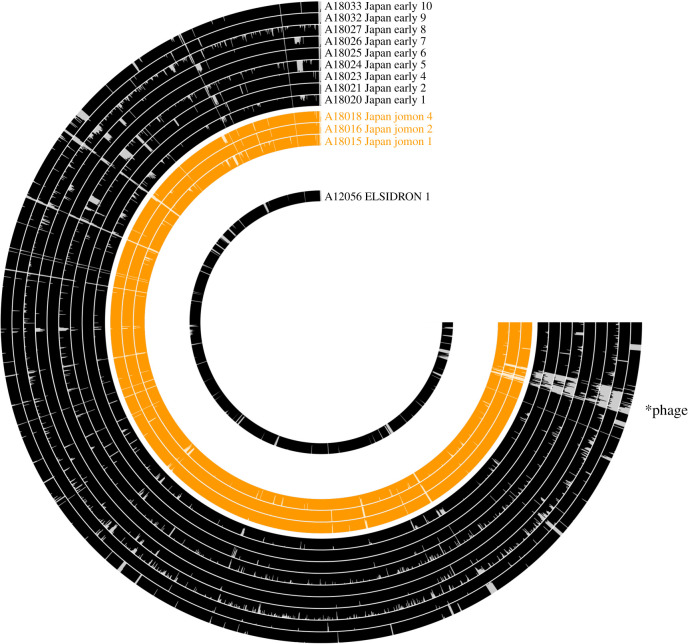
Read mapping results against the *Anaerolineaceae* sp*. oral taxon 439* genome. Each bin represents a gene, with the magnitude of the bar representing the mean depth of coverage of that gene (maximum 3). *Putative phage region that is present in modern reference.

**Figure 6.**
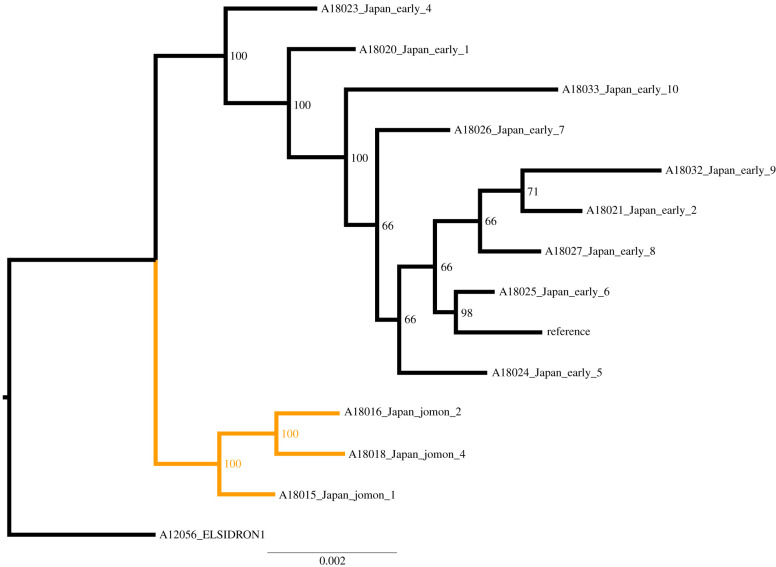
Maximum-likelihood phylogenetic tree of reconstructed *Anaerolineaceae* sp. *oral taxon 439* genomes. Node labels represent percentage support of 300 bootstrap replicates. Elsidron 1 Neanderthal set as an outgroup, with two separate clades containing Jomon (orange), or Edo period Japanese samples (black). Elsidron1 = Elsidron Neanderthal, Reference genome = *Anaerolineaceae* sp. *oral taxon 439* (ASM171754v1).

We then used a conservative approach to examine *Anaerolineaceae* genomic variants in all individuals. DNA sequences mapped evenly across the genome (figure 5), and whole-genome phylogenetic reconstruction found strong support for a distinct Jomon clade (figure 6), which clustered separately from Edo period samples (figure 6). This suggests that at least two distinct lineages of *Anaerolineaceae* strains existed in ancient Japan.

## 4. Discussion

This study is, to our knowledge, the first to explore oral microbiomes from ancient Japanese individuals, providing evidence for past microbial changes in response to disease and changes in human demography. While we did not observe major differences between Jomon and Edo period microbiome compositions, differences between male and female Edo period Japanese individuals were apparent, although the contributions of cultural practices and periodontal disease need further investigation. Finally, phylogenomic investigations revealed at least two distinct *Anaerolineaceae* sp. lineages between Jomon and Edo periods.

The switch to agricultural lifestyles from hunting and gathering has been associated with a compositional change in oral microbiota [28,30]. Here, we assessed dental calculus in both hunter–gatherers and agriculturalists in ancient Japan. Archaeological evidence suggests that Jomon hunter–gatherers relied on both terrestrial and marine resources, including nuts, deer, boar, marine fishes and shellfish [92]. Carbon isotope ratios of human teeth also suggest that C3 plants and terrestrial mammals were major dietary resources for the Jomon people [41]. This is in stark contrast to individuals from the Edo period, who led a predominantly agricultural subsistence [43]. Contrary to published research conducted in Europe, we did not detect a statistically significant difference in microbiome composition between Jomon (hunter–gatherer) and Edo (agricultural) period Japanese. Furthermore, no microbial species were found to be differentially abundant between cultures, which could suggest that the classifiable oral microbiome composition did not drastically change in Japan from Jomon to Edo periods. This supports findings that oral microbiota are highly stable through time [93,94] and maybe minimally influenced by certain dietary changes [95,96]. This is also consistent with other studies comparing modern hunter–gatherer populations to industrialized populations, which failed to see large changes in oral microbiota between these two lifestyles, despite findings changes in gut microbiota [16]. However, there are several alternative explanations. First, the limited sample size of our study (four Jomon and 10 Edo period) could have prevented the detection of such differences. Additionally, we were not able to control for tooth type owing to our small sample size, which has been shown to influence the microbial composition of modern plaque [97] and ancient dental calculus samples [98]. Bioinformatically, we may have also experienced biases in our species identifications, as the species that we classified maybe biased towards core oral taxa that are stable through time in Europeans, given that most modern oral reference genomes are more commonly generated from European and American isolates [93,94,97,99]. Furthermore, we were only able to classify on average approximately 49.8% of sequences from the ancient Japanese samples, consistent with other ancient calculus studies (e.g. [51]), and therefore, perhaps missed some of the microbial diversity present in these ancient samples that were unique to each culture or labile to dietary changes. Future improvements of analytical tools and further sampling of oral microbial genomes from broader human populations could allow for classification of the unclassified portion of our data and potentially provide enhanced bio-archaeological information from ancient dental calculus.

We found a significant difference between the microbiome composition of female and male Edo period Japanese. A potential driver of this difference is oral disease status, as all of the female samples had evidence of periodontal disease, which has been demonstrated in modern populations to impact microbiome composition [85,87,100]. In particular, we found the periodontal disease-associated archaeon, *Methanobrevibacter oralis* [77,89,90], to be generally more abundant in females versus males Edo period Japanese, although members of the periodontitis-associated ‘red-complex’ were not found to be differentially abundant in females versus males [88]. However, this is unsurprising given recent recognition that periodontal disease is of complex aetiology, not the result of a handful of periopathogens [101]. Interestingly, one Edo period sample (A18022) was also compositionally distinct from others and had 10 species classified that were not found in any other sample. This sample clustered with the modern healthy HMP plaque samples on the PCoA plot and in SourceTracker analysis and could represent a ‘healthy’ ancient sample. Future studies with larger sample sizes including both periodontal-positive and negative individuals are needed to determine the influence of periodontal disease on the male/female split we observed in Edo period Japanese. Overall, our findings suggest that periodontal disease is an important factor to examine when comparing microbial composition in ancient dental calculus studies, and future studies should aim to control for periodontal disease when making cultural comparisons.

It is also possible that the use of Ohaguru may have also influenced the female oral microbiota in ancient Japan. The practice of Ohaguru was common in higher-status women until the end of the Edo period, when the practice was outlawed in 1870 [102]. Women would paint their teeth with a black paste, called kanemizu, which was typically a mixture of iron (ferric) acetate and vegetable tannins; for example, kanemizu can be created by dissolving iron filings in vinegar and then adding in tannins from tea [103]. It is plausible that introduction of both iron and vegetable tannins using this method influenced the oral microbiota. For example, iron availability in an *in vitro* model of salivary microbiota had a significant impact on the microbiota composition [104]. Further, tea drinking has been shown to significantly alter both salivary oral microbiota and that surveyed by systematic oral brushing [105,106]. Regardless, this practice probably impacted the oral microbiota via access to these micronutrients, as previously reported isotope data from the skeletons found no significant differences in the general dietary intake between male and female samples from the Early Edo period [43]. Surprisingly, the practice of Ohaguru was thought to protect teeth from dental decay [103]; however, we find it associated with evidence of periodontal disease, raising questions about its health benefits. Future studies could empirically test the impact of kanemizu on oral microbiota using *in vitro* models or examining the impact of other tooth blackening processes on oral microbiota and health, as tooth blackening was practised historically and is still practised today in Oceania [107]. Nevertheless, microbiome studies may provide further information into how cultural practices influenced oral health in the past and today.

It is widely accepted that the modern Japanese population is the result of admixture between indigenous Jomon and later migrants from continental Asia during and after the Yayoi period [37]. Here, we observed a separation between Edo-associated *Anaerolineaceae* lineages and those found in ancient Jomon samples. While it is unclear how these two distinct clades originated, one potential hypothesis is that the Edo-period *Anaerolineaceae* strain originated in Japan through human demographic processes. For example, continental Asian *Anaerolineaceae* lineage/s could have been brought to Japan by migrants who arrived in Japan from mainland Asia. It also remains unclear if either strain still exists today, or if the prevalence of the Jomon-period strain was diminished by the Edo strain. This later scenario is plausible if the continental Asian contribution to modern Japanese was larger than the Jomon, resulting in the loss of the lineage in a fashion analogous to genetic drift. Current estimates of Jomon genetic contribution to modern Japanese is less than 20%, supporting this scenario [37]. However, another possibility for this finding is that the Jomon lineage has survived to this day, but that we did not detect it owing to the small sample size of our study and lack of comparable modern metagenomic data. Future studies investigating modern individuals from across Japan could test for the presence of the Jomon *Anaerolineaceae* lineage and try to pinpoint the source of the Edo strains. Spatially diverse sampling will be important, as it has been shown that genetic contribution from Jomon varied among populations across the Japanese Archipelago [35–37]. Further studies using ancient dental calculus could also assist in learning more about the source/s of Yayoi admixture, or the diversity of Jomon strains prior to the arrival of mainland migrants, which remain undetermined. Future DNA sequencing efforts will allow for the phylogenetic reconstruction of other human-associated microorganisms and permit investigations into how these genomes have changed through time, potentially yielding insights into mechanisms of co-speciation with humans.

## Supplementary Material

Supplementary tables S1-S7

## Supplementary Material

Supplementary figures S1-S8

## Supplementary Material

BED file containing phage, 16S rRNA and rRNA gene regions masked in the Anaerolineaceae sp. oral taxon 439 alignments.

## Data accessibility

The untrimmed, demultiplexed paired-end sequences generated in this study are available at the NCBI SRA under project PRJNA608555
