# Supplementary material for: Investigating the demographic history of Japan using ancient oral microbiota
Source: Philos Trans R Soc Lond B Biol Sci. 2020 Oct 5;375(1812):20190578. doi: 10.1098/rstb.2019.0578 (PMC7702792; doi:10.1098/rstb.2019.0578)
Supplement: Supplementary figures S1-S8 [file rstb20190578supp2.pdf]

Figure S1. Taxonomic profile for 18019_Japan_Jomon_5. Taxa coloured red are putative contaminants.

Figure S2. MapDamage cytosine deamination plots of reads mapping to the Anaerolineaceae sp oral taxon 439 genome. The left and right panels for each sample display the 5' C-to-T (red lines) and 3' G-to-A (blue lines) substitution rates, respectively.

Figure S3. MapDamage cytosine deamination plots of reads mapping to the Anaerolineaceae sp oral taxon 439 genome. The left and right panels for each sample display the 5' C-to-T (red lines) and 3' G-to-A (blue lines) substitution rates, respectively.

Figure S4. Edit distances of reads from samples aligned to the Anaerolineaceae sp oral taxon 439 genome.

Figure S5. Edit distances of reads from samples aligned to the Anaerolineaceae sp oral taxon 439 genome.

Figure S6. Edit distances of reads from samples aligned to the Anaerolineaceae sp oral taxon 439 genome.

Figure S7. Edit distances of reads from samples aligned to the Anaerolineaceae sp oral taxon 439 genome.

Figure S8. SourceTracker analysis of ancient dental calculus samples.

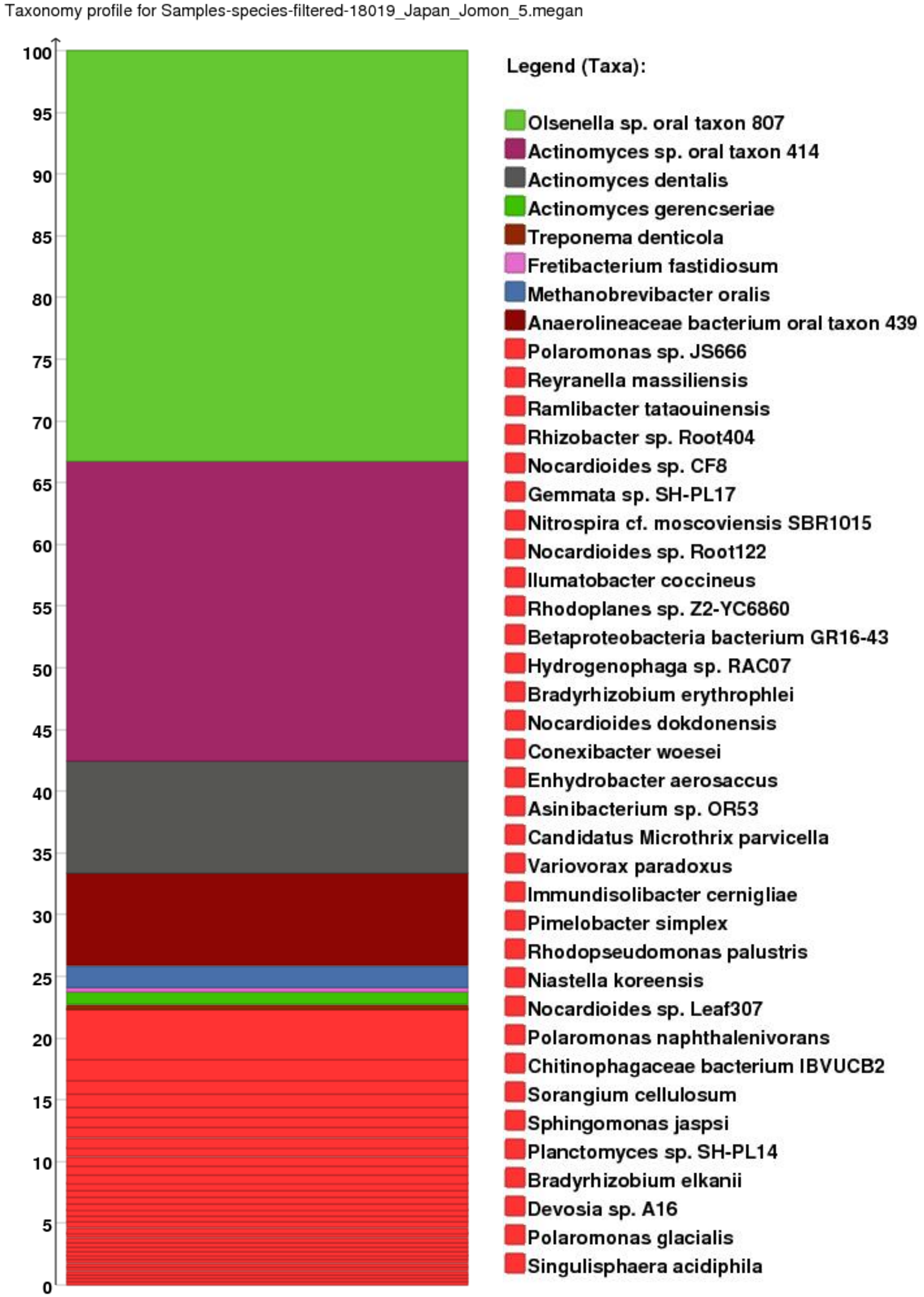

Figure S1: Taxonomic profile for 18019_Japan_Jomon_5. Taxa coloured red are putative contaminants.

Figure S2

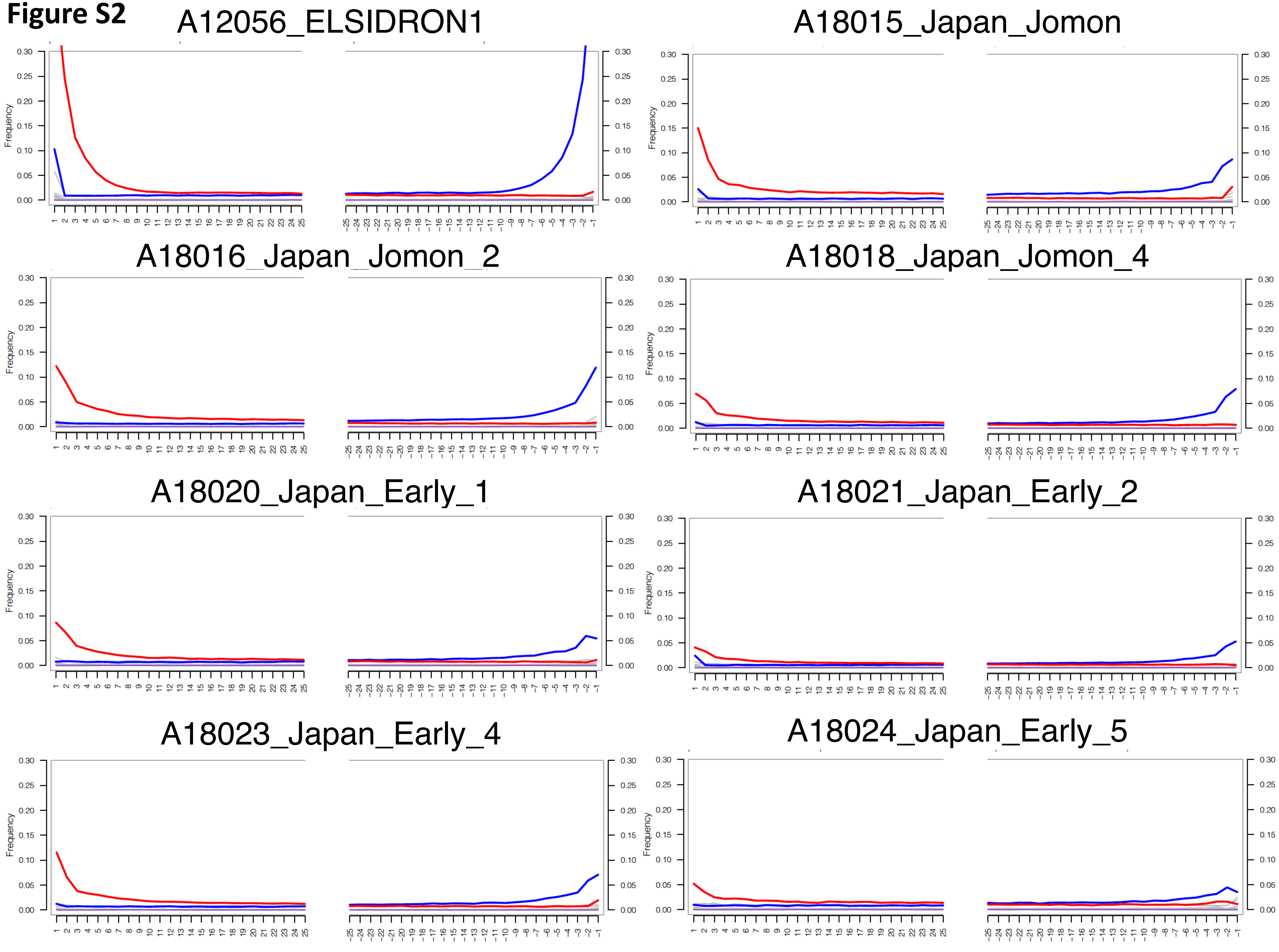
A12056_ELSIDRON1
A18015_Japan_Jomon
A18016_Japan_Jomon_2
A18018_Japan_Jomon_4
A18020_Japan_Early_1
A18021_Japan_Early_2
A18023_Japan_Early_4
A18024_Japan_Early_5
Frequency

Figure S3

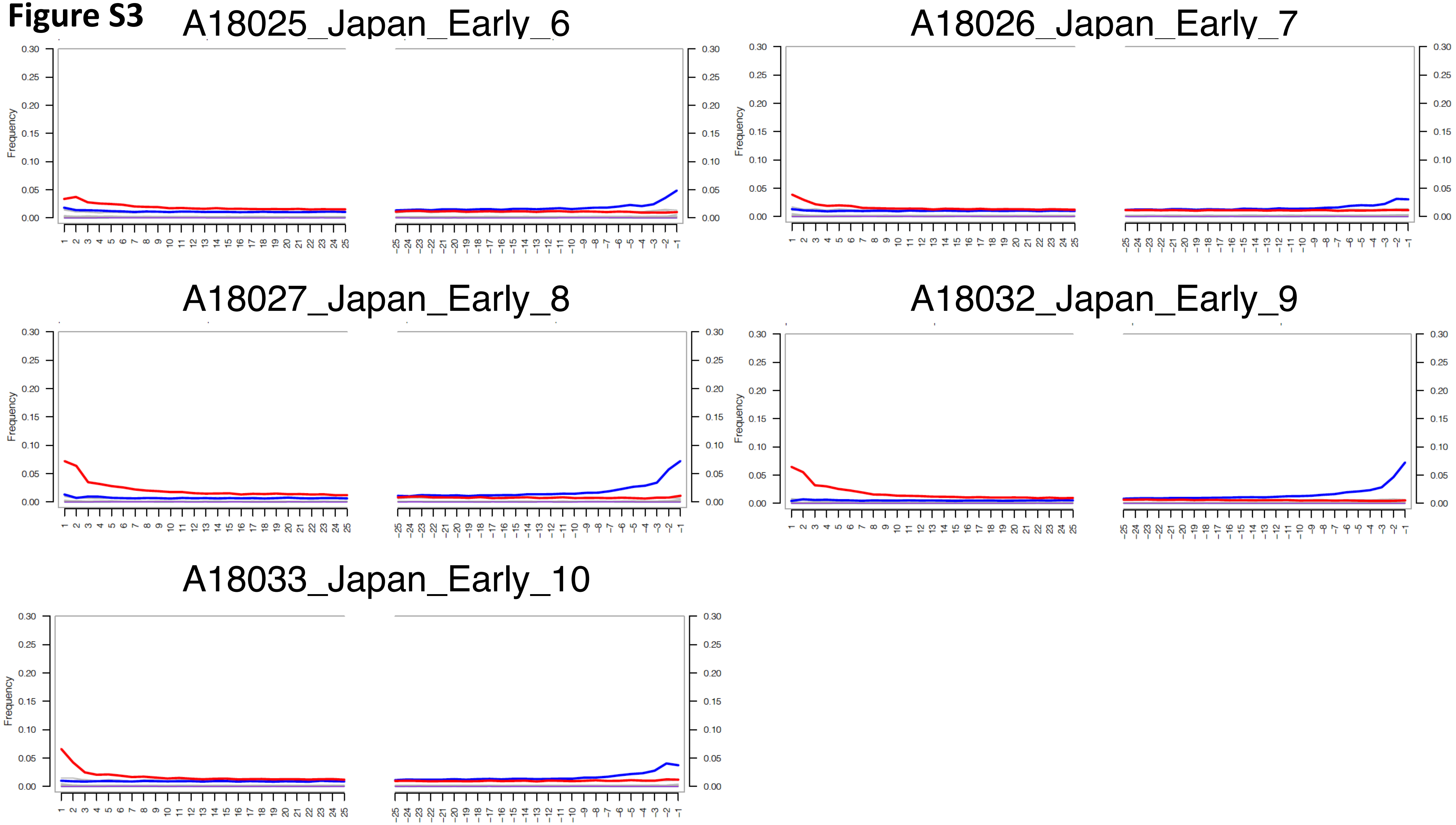
A18025_Japan_Early_6
A18026_Japan_Early_7
A18027_Japan_Early_8
A18032_Japan_Early_9
A18033_Japan_Early_10
Frequency
0.00 0.05 0.10 0.15 0.20 0.25 0.30
1 2 3 4 5 6 7 8 9 10 11 12 13 14 15 16 17 18 19 20 21 22 23 24 25
-25 -24 -23 -22 -21 -20 -19 -18 -17 -16 -15 -14 -13 -12 -11 -10 -9 -8 -7 -6 -5 -4 -3 -2 -1

Figure S4

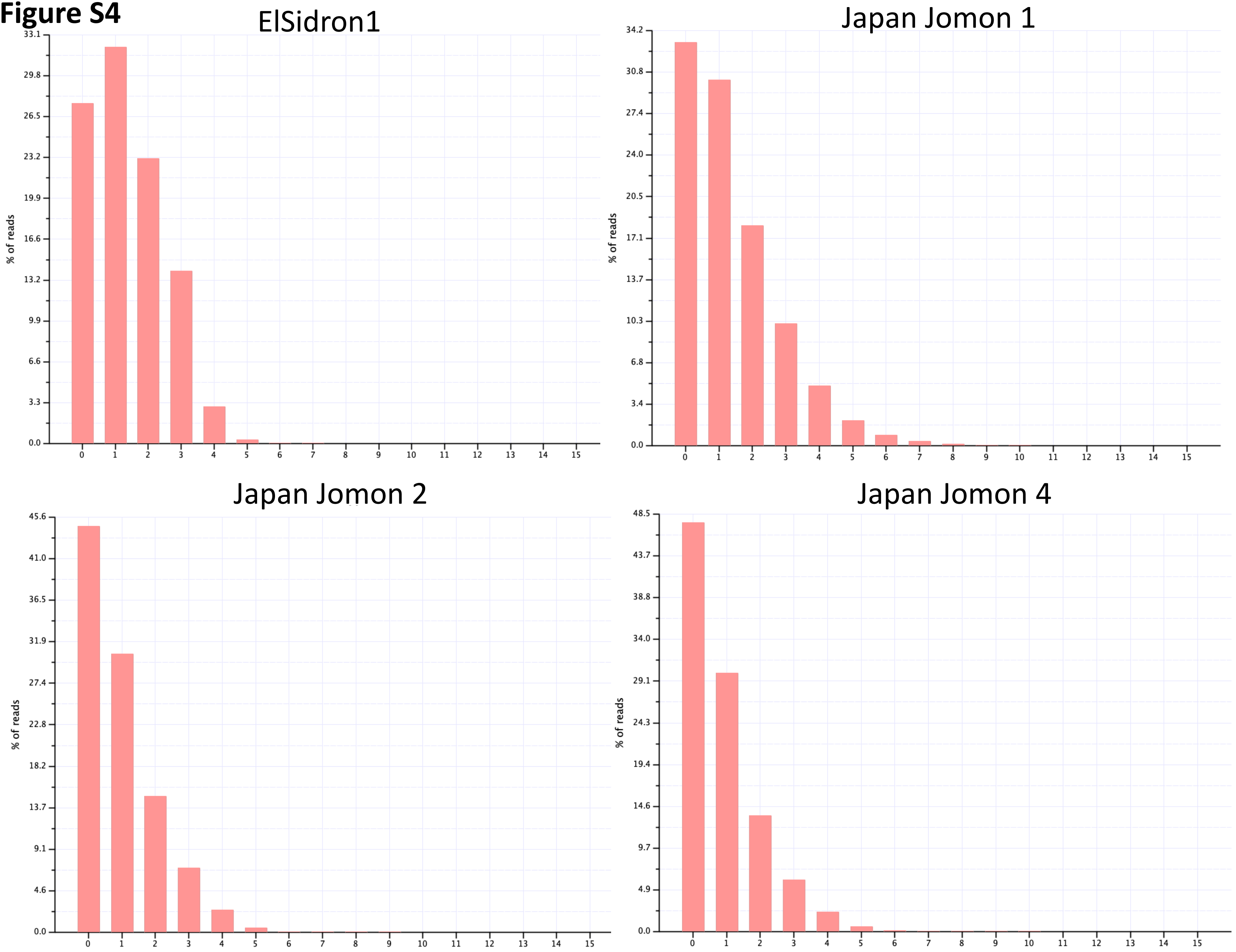

ElSidron1
Japan Jomon 1
Japan Jomon 2
Japan Jomon 4
% of reads
33.1
29.8
26.5
23.2
19.9
16.6
13.2
9.9
6.6
3.3
0.0
34.2
30.8
27.4
24.0
20.5
17.1
13.7
10.3
6.8
3.4
45.6
41.0
36.5
31.9
22.8
18.2
9.1
4.6
48.5
43.7
38.8
34.0
29.1
24.3
19.4
14.6
9.7
4.9
0 1 2 3 4 5 6 7 8 9 10 11 12 13 14 15

Figure S5

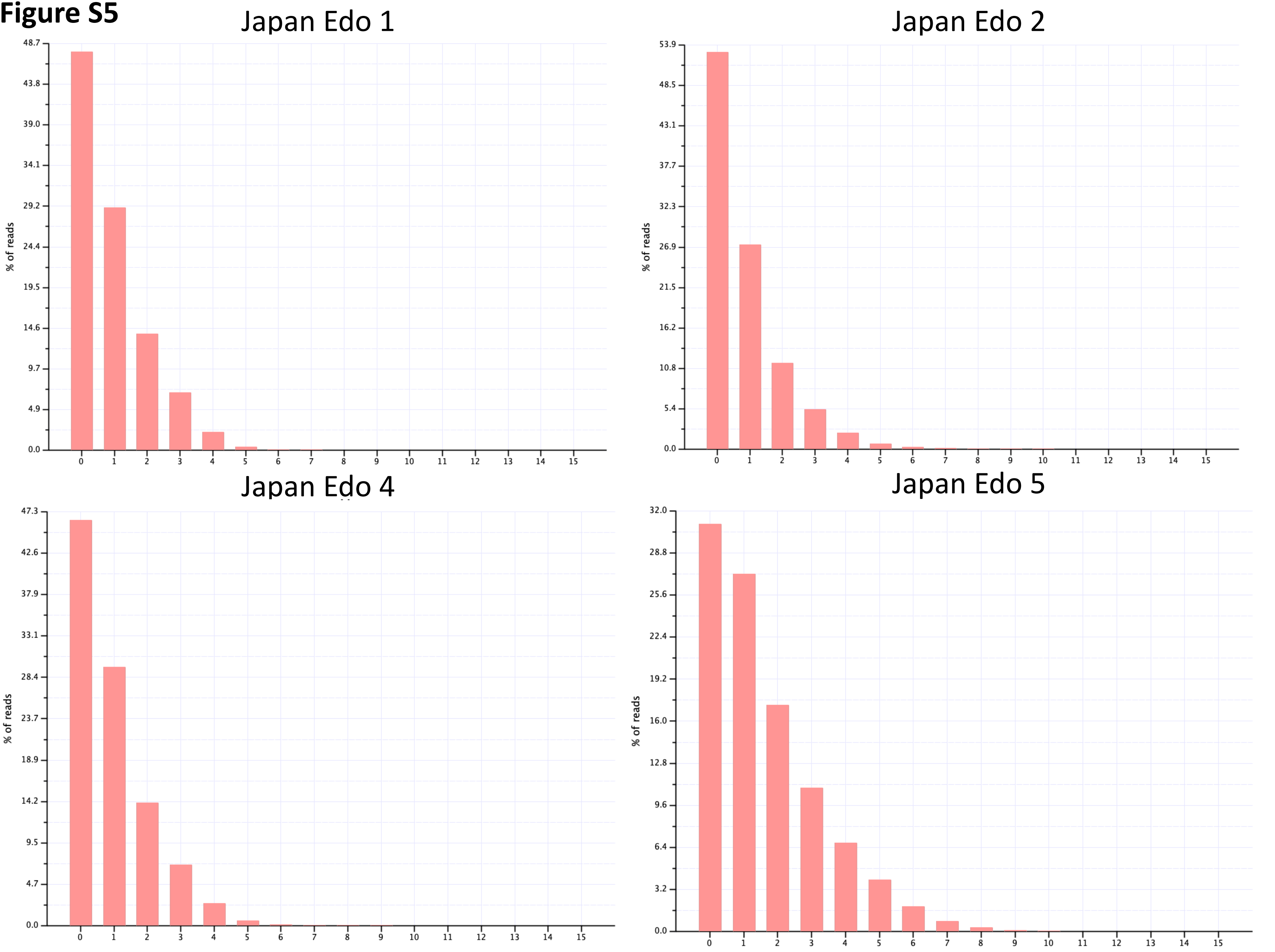

Japan Edo 1
% of reads
48.7
43.8
39.0
34.1
29.2
24.4
19.5
14.6
9.7
4.9
0.0
0 1 2 3 4 5 6 7 8 9 10 11 12 13 14 15
Japan Edo 2
% of reads
53.9
48.5
43.1
37.7
32.3
26.9
21.5
16.2
10.8
5.4
0.0
0 1 2 3 4 5 6 7 8 9 10 11 12 13 14 15
Japan Edo 4
% of reads
47.3
42.6
37.9
33.1
28.4
23.7
18.9
14.2
9.5
4.7
0.0
0 1 2 3 4 5 6 7 8 9 10 11 12 13 14 15
Japan Edo 5
% of reads
32.0
28.8
25.6
22.4
19.2
16.0
12.8
9.6
6.4
3.2
0.0
0 1 2 3 4 5 6 7 8 9 10 11 12 13 14 15

Figure S6

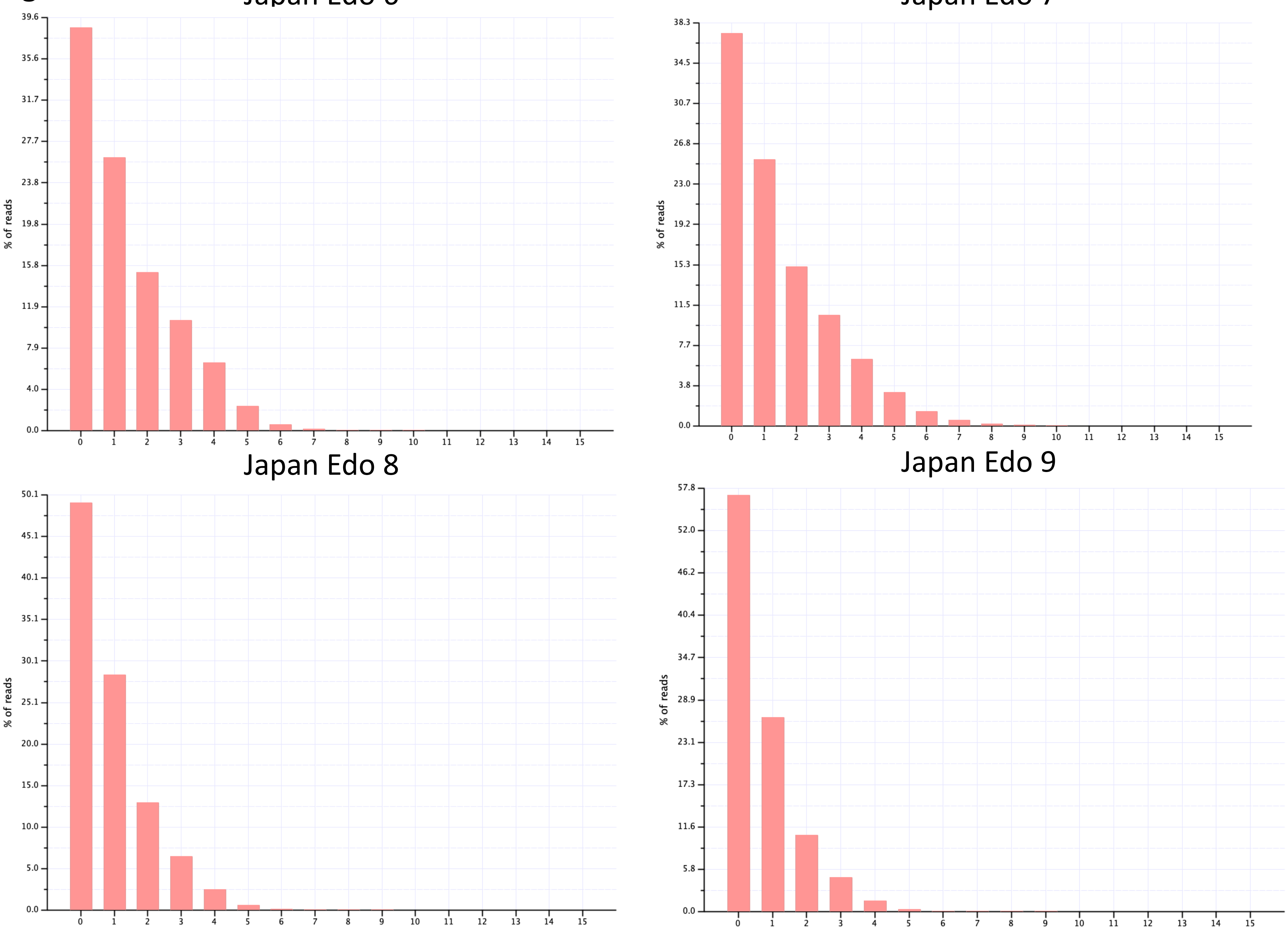
Japan Edo 6
Japan Edo 7
Japan Edo 8
Japan Edo 9
% of reads
0.0 4.0 7.9 11.9 15.8 19.8 23.8 27.7 31.7 35.6 39.6
0.0 3.8 7.7 11.5 15.3 19.2 23.0 26.8 30.7 34.5 38.3
0.0 5.0 10.0 15.0 20.0 25.1 30.1 35.1 40.1 45.1 50.1
0.0 5.8 11.6 17.3 23.1 28.9 34.7 40.4 46.2 52.0 57.8
0 1 2 3 4 5 6 7 8 9 10 11 12 13 14 15

Figure S7

Japan Edo 10

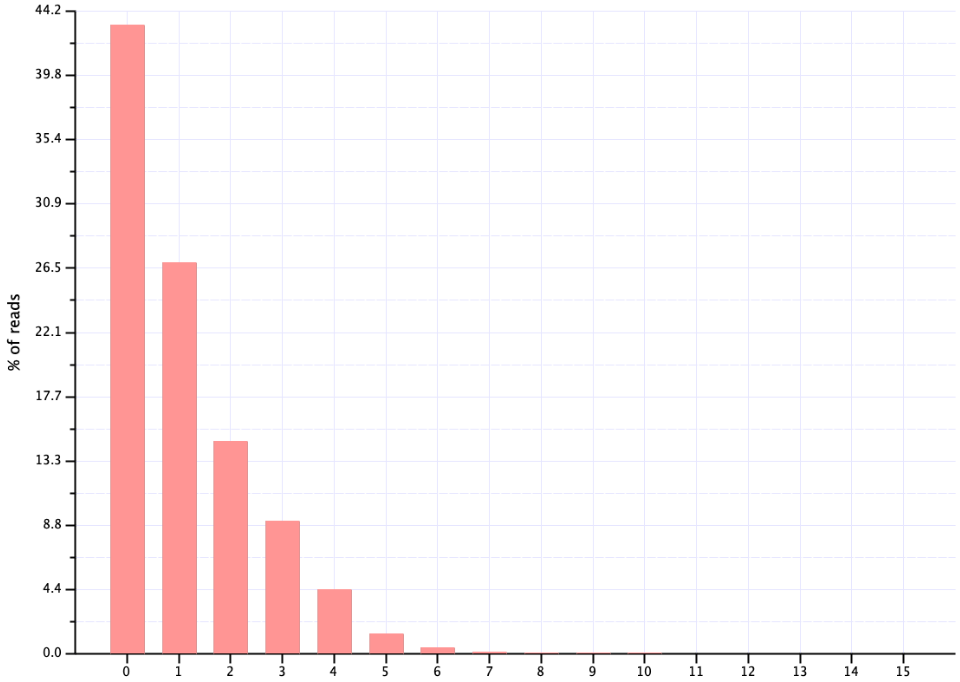

Figure S8

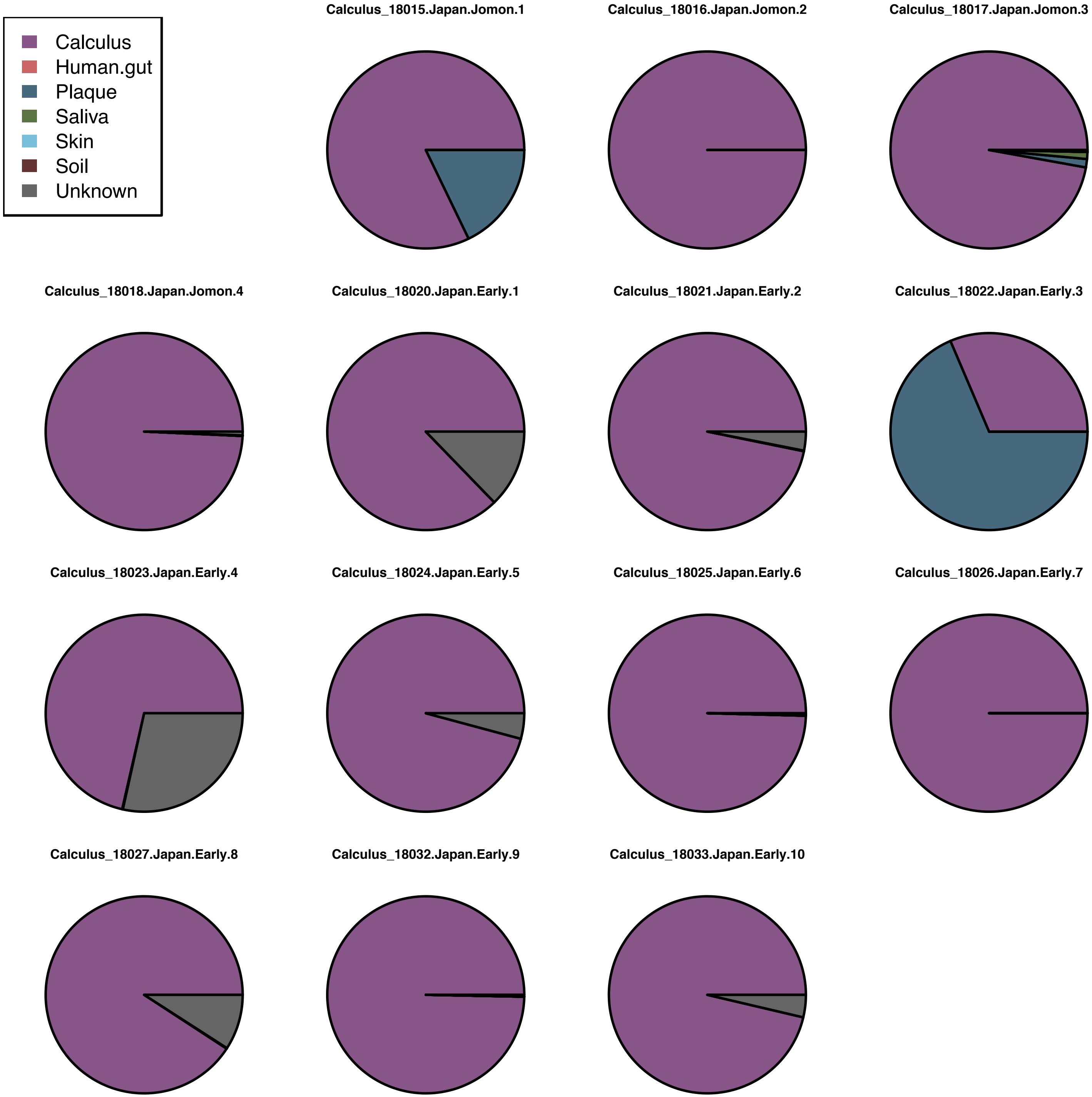
Calculus
Human.gut
Plaque
Saliva
Skin
Soil
Unknown
Calculus_18015.Japan.Jomon.1
Calculus_18016.Japan.Jomon.2
Calculus_18017.Japan.Jomon.3
Calculus_18018.Japan.Jomon.4
Calculus_18020.Japan.Early.1
Calculus_18021.Japan.Early.2
Calculus_18022.Japan.Early.3
Calculus_18023.Japan.Early.4
Calculus_18024.Japan.Early.5
Calculus_18025.Japan.Early.6
Calculus_18026.Japan.Early.7
Calculus_18027.Japan.Early.8
Calculus_18032.Japan.Early.9
Calculus_18033.Japan.Early.10
